# Levetiracetam Differentially Alters CD95 Expression of Neuronal Cells and the Mitochondrial Membrane Potential of Immune and Neuronal Cells *in vitro*

**DOI:** 10.3389/fneur.2014.00017

**Published:** 2014-02-18

**Authors:** Susannah K. Rogers, Lee A. Shapiro, Richard P. Tobin, Benjamin Tow, Aleksej Zuzek, Sanjib Mukherjee, M. Karen Newell-Rogers

**Affiliations:** ^1^Department of Anthropology, University of Texas, Austin, TX, USA; ^2^Department of Surgery, Texas A&M University Health Science Center, Temple, TX, USA; ^3^Central Texas Veterans Health Care System, Temple, TX, USA; ^4^Scott and White Hospital, Temple, TX, USA

**Keywords:** epilepsy, Keppra, splenocytes, C17.2, *in vitro*, Fas, FasL

## Abstract

Epilepsy is a neurological seizure disorder that affects over 100 million people worldwide. Levetiracetam, either alone, as monotherapy, or as adjunctive treatment, is widely used to control certain types of seizures. Despite its increasing popularity as a relatively safe and effective anti-convulsive treatment option, its mechanism(s) of action are poorly understood. Studies have suggested neuronal, glial, and immune mechanisms of action. Understanding the precise mechanisms of action of levetiracetam would be extremely beneficial in helping to understand the processes involved in seizure generation and epilepsy. Moreover, a full understanding of these mechanisms would help to create more efficacious treatments while minimizing side-effects. The current study examined the effects of levetiracetam on the mitochondrial membrane potential of neuronal and non-neuronal cells, *in vitro*, in order to determine if levetiracetam influences metabolic processes in these cell types. In addition, this study sought to address possible immune-mediated mechanisms by determining if levetiracetam alters the expression of immune receptor–ligand pairs. The results show that levetiracetam induces expression of CD95 and CD178 on NGF-treated C17.2 neuronal cells. The results also show that levetiracetam increases mitochondrial membrane potential on C17.2 neuronal cells in the presence of nerve growth factor. In contrast, levetiracetam decreases the mitochondrial membrane potential of splenocytes and this effect was dependent on intact invariant chain, thus implicating immune cell interactions. These results suggest that both neuronal and non-neuronal anti-epileptic activities of levetiracetam involve control over energy metabolism, more specifically, mΔΨ. Future studies are needed to further investigate this potential mechanism of action.

## Introduction

Epilepsy is a neurological disorder that affects approximately 65 million people worldwide. While numerous treatment options are available to control seizures associated with epilepsy, approximately 20–30% of epileptic patients are resistant to treatment. Pharmaceutical and biomedical device companies continue to develop new treatments that are more efficacious, while minimizing undesirable side-effects. One such drug, levetiracetam, alone as a monotherapy, or combined with another treatment as an adjunct, is widely used to control partial onset and generalized seizures (1). Despite its use as a relatively safe and effective anti-convulsant treatment option, the precise mechanism(s) of action are not fully understood.

The anti-epileptic effects of levetiracetam may occur, at least in part, by acting directly on neurons. For example, levetiracetam is known to bind to the synaptic vesicle protein SV2A (2) and to inhibit presynaptic calcium channels (3). Studies have shown that levetiracetam may also exert its anti-epileptic effects by reducing calcium currents in CA3 pyramidal neurons of the hippocampus (4), or by rescuing neurons in the hippocampus and dentate gyrus from death (5). Another possibility is that levetiracetam alters mitochondrial membrane potential (mΔΨ), although the reports using the *in vivo* perforant pathway stimulation paradigm are conflicting. In one study, levetiracetam effectively mitigated mitochondrial dysfunction in the hippocampus following established *status epilepticus* (6), but not at acute time points after the onset of *status epilepticus* (7). Thus, it is unclear if, or to what extent, the therapeutic effects of levetiracetam can be attributed to its neuronal interactions.

While the purpose of the current study is to determine if there are direct effects of levetiracetam on neuronal and immune cells, other studies have suggested glial cell mechanisms of action. For example, Ueda et al. (8) suggested that levetiracetam exerts its neuroprotective effects through its actions on glial cells. Similarly, Haghikia et al. (9) showed that levetiracetam has anti-inflammatory effects on astrocytes. Consistent with this finding, Kim et al. (10) showed that levetiracetam reduced gliosis in epileptic brains and inhibited IL-1B, and Stienen et al. (11) showed that the anti-inflammatory effects of levetiracetam on astrocytes may be mediated by TGFβ1.

In addition to effects in the CNS, levetiracetam could also be exerting its effects in the periphery. Supporting this notion are the results of a previous study demonstrating that levetiracetam inhibits the function of some CD8^+^ T Lymphocytes (12). Such interactions with the peripheral immune system might explain the increased incidence of pharyngitis and rhinitis in levetiracetam-treated patients (13–18). However, studies are lacking that provide a thorough analysis of the effects of levetiracetam on peripheral immune cells.

Understanding the mechanism(s) of action of levetiracetam is important because this knowledge could lead to more efficacious treatments and better understanding of the epileptic condition. Due to the lack of a unified theory for the mechanism(s) of action, the current study was designed to determine if levetiracetam affects the mΔΨ of peripheral immune cells and neuronal cells. Moreover, this study sought to address possible immune-mediated mechanisms by determining if levetiracetam alters the expression of immune receptor–ligand pairs.

## Materials and Methods

### Cell lines

#### C17.2

The C17.2 cell line is an immortalized mouse neural progenitor cell line capable of differentiation *in vitro*. The cell line was established by retroviral-mediated transduction of the avian *myc* oncogene into mitotic progenitor cells of neonatal mouse cerebellum from a CD1 × C57BL/6 mouse. The C17.2 line of neural stem cells responds to NGF by differentiating into more mature neuronal phenotypes and has been used extensively to monitor developmental regulation of mouse neurons (19). We employed this cell line as a model of mouse neuronal cells.

#### In vitro stimulations

C17.2 cells were either untreated, or treated with nerve growth factor (NGF) at 0.4 nM final concentration. All cells were treated with levetiracetam or vehicle for 48 h, at the following concentrations: 0.5 μm, 15 μm, 0.15 mM, or 1.5 mM.

#### Mice

Eight- to ten-week-old C57BL/6J male mice were purchased from Jackson Labs. Invariant chain (CD74)-deficient mice (Ii^Def^) (C57BL/6 background) were purchased from Jackson Labs and bred at the Scott and White Healthcare animal facility to maintain homozygosity. Mice were housed in the Scott and White Healthcare animal facility according to IACUC regulations.

#### Spleen cell isolation

Mice were sacrificed and spleens were removed. Splenocytes were dissociated by passing spleens through 40 μm cell strainers. Red blood cells were lysed using GEY’S buffer (20). Cells were then cultured at 1.010^6^ cells/mL in 6 well plates. Cells were grown in RPMI 1640 (Invitrogen) supplemented with 5% fetal bovine serum (Invitrogen) in a humidified 5% CO_2_ incubator at 37°C for the designated time period. Splenocytes were then treated with levetiracetam or vehicle for 48 h, at the following concentrations: 0.5 μm, 15 μm, 0.15 mM, or 1.5 mM.

#### Flow cytometry

For cell surface markers the cells were first blocked with FC Block (BD Bioscience) and then stained with the following antibodies; MHCII, CD3ε, CD80, CD86, Fas (CD95), and CD178 (BD Bioscience). Cells were analyzed using a BD FACS Canto II flow cytometer and the data was analyzed using FlowJo software (TreeStar Inc.).

#### Mitochondrial membrane potential (MΔΨ)

To assess the possibility that levetiracetam has direct effects on mitochondrial function, mitochondrial activity was assessed using MitoTracker Red CM-H2XRos (Life Technologies), a mitochondrial dye that fluoresces as a function of mΔΨ. Tightly regulated mΔΨ is essential for maintaining physiological function(s), including appropriate mitochondrial substrate selection for generating ATP and for maintaining cell viability. Cells were treated with MitoTracker Red and allowed to incubate in the dye for 20 min prior to analysis using a BD FACS Canto II flow cytometer. The flow cytometer measures mean fluorescent intensity per cell. Cells were untreated, treated with NGF, treated with levetiracetam or NGF + levetiracetam, as described above in the *in vitro* stimulation. For each treatment group, a minimum of four separate assays were performed in triplicate.

#### Lysosomal acidity

To assess the effects of levetiracetam on lysosomal pH, we used the fluorescent dye Lysosensor Green (Life Technologies). Lysosensor Green produces increased fluorescence intensity at lower pH. Cells were analyzed using a BD FACS Canto II flow cytometer.

#### Statistical analysis

Statistical analysis was performed using GraphPad Prism 6 (GraphPad Software Inc.). For comparisons between splenocytes from C57BL/6J and Ii^Def^, a paired *t*-test was used with a significance cut-off of *P* < 0.05. For all other analysis, repeated measures ANOVA was used with *post hoc* planned comparisons using Dunnett’s correction factor.

## Results

**Figure 1:**
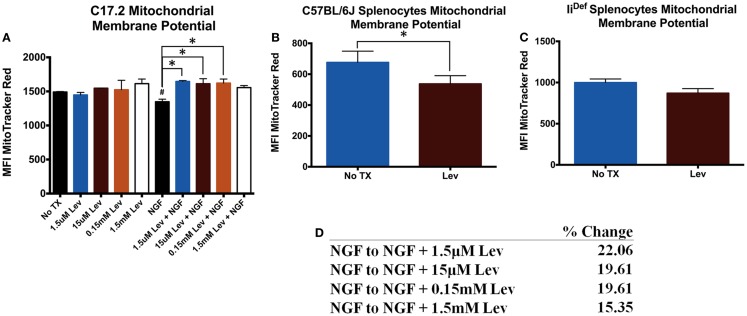
**Changes in mitochondrial membrane potential following treatment with levetiracetam**. **(A)** Mean fluorescence intensity (MFI) of Mitotracker Red as a measure of relative mitochondrial membrane potential in C17.2 cells at 48 h post treatment with NGF with or without levetiracetam (Lev). **(B)** MFI Mitotracker Red in C57BL/6 splenocytes 48 h after treatment with or without 0.15 mM Lev. **(C)** MFI Mitotracker Red in in Ii^Def^ splenocytes 48 h after treatment with or without 0.15 mM Lev. **(D)** Table depicting percent change from NGF treatment alone, compared to NGF treatment in the presence of doses of levetiracetam. *Denotes a *p*-value < 0.05.

Previous studies have indicated mitochondrial differences in the presence of levetiracetam (6, 7). Therefore, we determined if these differences were specific for neuronal or immune cells. Analysis of mΔΨ in C17.2 cells revealed no significant differences in the absence of NGF (Figure 1A). In the presence of NGF, levetiracetam resulted in a significant increase (Figure 1A) in mΔΨ at all concentrations tested (1.5 μm. *p* < 0.03; 15 μm, *p* < 0.05; 0.15 mM, *p* < 0.04; 1.5 mM, NS). It is pertinent to note that treatment with levetiracetam did not cause any observable alterations to the morphology of the C17.2 cells, either with or without NGF (data not shown). In contrast to the increased mΔΨ in the presence of NGF and levetiracetam, the impact of levetiracetam on spleen cells (Figure 1B) was a significant reduction in mΔΨ (*p* < 0.007). This reduction appeared to be invariant chain dependent, as splenocytes from mice deficient in invariant chain showed no significant changes in mΔΨ in response to levetiracetam (Figures 1C,D).

**Figure 2:**
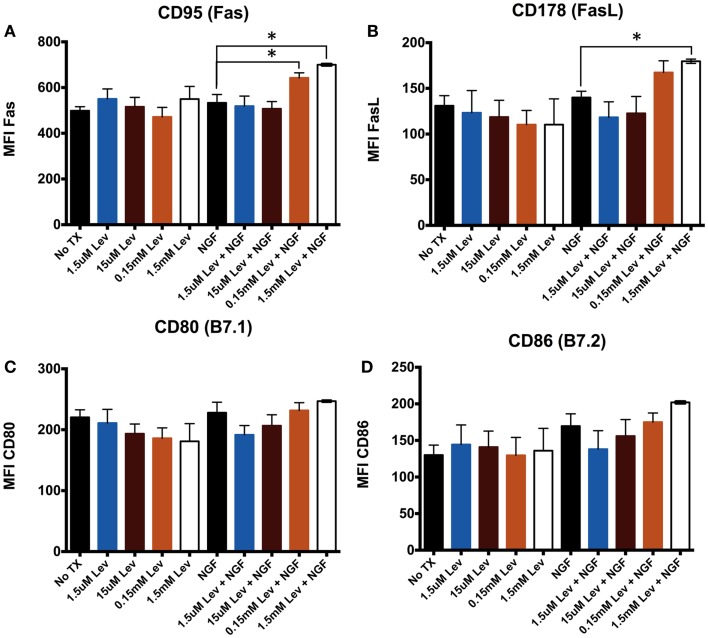
**Levetiracetam alters cell surface Fas expression on C17.2 cells**. Mean flouresecence intensity (MFI) as measure of relative expression level of **(A)** CD95, **(B)** CD178, **(C)** CD80, and **(D)** CD86 48 h after treatment with or without Lev. *Denotes a *p*-value < 0.05.

Elevated mΔΨ can be associated with elevated CD95 (21, 22). Therefore, to address the possibility that levetiracetam alters receptor–ligand pairs on neurons, we used the mouse neuronal stem cell line, C17.2, which can be differentiated in the presence of NGF. We assessed CD95, a member of the BGF superfamily and its ligand, FasL (CD178) to determine if levetiracetam can influence cell proliferation, differentiation, and survival. We also examined alterations in the co-stimulatory molecules B7.1 (CD80), or B7.2 (CD86) to assess the potential of levetiracetam to alter co-stimulation of T cell activation. The results from analysis of C17.2 cells revealed that levetiracetam treatment alone had no significant effects on CD95 (Figure 2A), CD178 (Figure 2B), CD80 (Figure 2C), or CD86 (Figure 2D). In the presence of NGF, no significant differences were observed for CD95, CD178, CD80, or CD86 at the 1.5 or 15 μm concentrations. However, at 0.15 and 1.5 mM, a significant increase in CD95 (*p* < 0.02 and *p* < 0.001, respectively) was observed. At these latter two concentrations, no significant differences were observed for CD80 or CD86. For CD178, no significant differences were observed for the three lowest concentrations of levetiracetam, but at the 1.5 mM concentration, a significant increase was observed for CD178 (*p* < 0.05).

**Figure 3:**
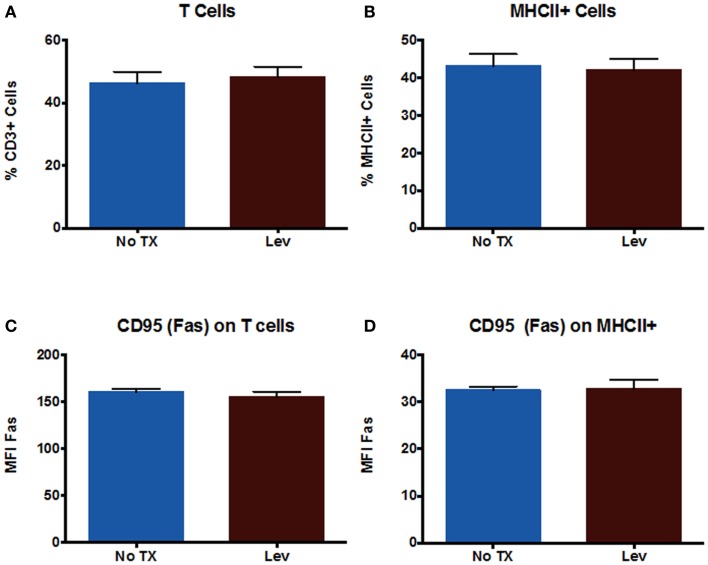
**Levetiracetam does not Alter Immune Cells *in vitro***. The percentage of **(A)** T cells and **(B)** MHCII^+^ cells in splenocytes 48 h after treatment with or without 0.15 mM Lev. Mean fluorescence intensity (MFI) as measure of relative expression level of CD95 on **(C)** T cells and **(D)** MHCII^+^ cells in splenocytes 48 h after treatment with or without 0.15 mM Lev. *Denotes a *p*-value < 0.05.

**Figure 4:**
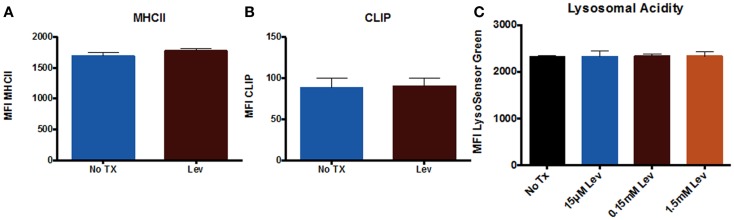
**Levetiracetam does not alter antigen processing and presentation machinery *in vitro***. Mean fluorescence intensity (MFI) as measure of relative expression level of **(A)** MHCII^+^ and **(B)** CLIP on splenocytes 48 h after treatment with or without Lev. **(C)** MFI Lysosensor Green as a relative measure of lysosomal acidity of splenocytes 48 h after treatment with Lev. *Denotes a *p*-value < 0.05.

In addition to examining neuronal cells, we also examined peripheral immune cells from the spleen. We examined numbers of T cells and numbers of MHCII^+^ cells (which includes macrophages and B cells), as well as CD95 expression on these cells. The results showed that levetiracetam treatment resulted in no significant effect on the number of CD3^+^ T cells (Figure 3A), MHCII^+^ (Figure 3B) cells, nor on the levels of CD95 expression by T cells (Figure 3C), and non-T cells (Figure 3D). In addition, we examined overall levels of MHCII and CLIP on non-T cells (Figures 4A,B) to address the possibility that levetiracetam alters immunogenicity of peripheral immune cells. No changes were observed for either of these variables (Figures 4A,B). To further detect levetiracetam-induced changes in processing or presentation by immune cells, we assessed lysosomal acidity and found no significant changes (Figure 4C).

## Discussion

Levetiracetam is well established as a beneficial anti-seizure medication and as an adjunct to other anti-seizure medications. The molecular mechanisms accounting for the efficacy of levetiracetam for seizure activity are largely unknown. The results from the present study suggest that levetiracetam induces expression of CD95 and CD178 on NGF-treated C17.2 neuronal cells. The results also demonstrate that the increased mΔΨ in response to levetiracetam on C17.2 neuronal stem cells requires the presence of NGF. This is likely due to the differentiating effect of NGF on neural stem cells. In contrast, the study shows that levetiracetam lowers the mΔΨ of splenocytes and this effect is dependent on intact invariant chain. These results suggest that both neuronal and non-neuronal anti-epileptic activities of levetiracetam involve control over energy metabolism, more specifically, mΔΨ.

Epilepsy has traditionally been considered primarily a neuronal disease. Growing evidence also implicates astrocytes, microglia, peripheral leukocytes, and blood–brain barrier breakdown in the pathogenesis of epilepsy. Here, we show two novel observations that potentially link peripheral leukocytes to neurons. Our results suggest that levetiracetam affects mitochondrial energy metabolism as reflected by changes in mΔΨ. Interestingly, these changes are inversely related when comparing splenocytes to NGF-treated neuronal stem cells. That is, levetiracetam causes a statistically significant increase in the membrane potential of NGF-treated C17.2 cells and a significant decrease in mΔΨ of levetiracetam-treated spleen cells. It is pertinent to note that in mice deficient for CD74, the levetiracetam-induced change to splenocyte mΔΨ was ameliorated. Thus, it is possible that levetiracetam-induced changes in mitochondrial activity result from cell–cell contact because CD74 can be expressed on the cell surface and mediate interactions with other cells through its cognate ligand, CD44. Alternatively, the requirement for CD74 to see the effects.

Previous studies have suggested that mitochondrial dysfunction contributes to the epileptic condition (23). The putative functional significance of levetiracetam-induced alterations to mΔΨ in the epileptic brain is its known effects on proton transport. Previous studies have demonstrated that alterations to the mΔΨ in neurons (24) and non-neuronal cells (25), directly influences ion concentrations in the cytosol, thereby influencing plasma membrane potential. Alterations to mΔΨ have also been shown to influence oxidative stress (26), which may be another anti-epileptic mechanism. A third potential mechanism through which altered mΔΨ could influence seizures is by altering the cytosolic pH. An acidification of cytosol as a result of protonation is known to hyperpolarize the plasma membrane (27), which may raise the seizure threshold. Support for this latter suggestion is observed in epileptic hippocampal slices where the pattern of epileptic activity corresponds to mΔΨ and ion concentration (23).

Previous work from our lab demonstrated that Fas/FasL interactions can facilitate neurite outgrowth subsequent to nerve crush injury (28). CD95 and its ligand CD178, a member of the NGF/NGF receptor superfamily of death-inducing receptor–ligand pairs. Many members of this superfamily are involved in cell fate decisions including cell death, cell proliferation, and differentiation. We addressed the possibility that if levetiracetam altered mitochondrial activity, it might also affect CD95 expression because elevated mΔΨ can be associated with elevated CD95. The results from the current study are consistent with this idea because we found that in neuronal cells, in the presence of NGF and >0.15 mM levetiracetam, mΔΨ is increased as is Fas expression. Therefore, levetiracetam may be involved in stabilizing mΔΨ in the presence of elevated levels of NGF.

Another potential effect of levetiracetam on leukocytes could be related to some of the side-effects associated with levetiracetam. In particular, an increased incidence of pharyngitis and rhinitis has been observed in levetiracetam-treated patients (13–18). These findings are consistent with a role for alterations to an effective immune response that may involve alterations in CD74 expression and function. A second possibility is related to the finding of reduced mΔΨ in splenocytes, which may reflect altered levels of immune function, including increased inflammation accounting for pharyngitis and rhinitis.

Overall, the data from the current study indicate that levetiracetam differentially affects the mΔΨ of neuronal C17.2 and non-neuronal splenocytes. The results also show that in the presence of elevated NGF, neuronal C17.2 cells express CD95 and CD178. The results from this study could help to explain some of the mechanisms of action of levetiracetam, including some of its side-effects. More studies are needed to better understand the implications of these findings so that more efficacious treatments with minimal side-effects can be developed.

## Conflict of Interest Statement

The authors declare that the research was conducted in the absence of any commercial or financial relationships that could be construed as a potential conflict of interest.

## References

[1] Surges R Volynski KE Walker MC Is levetiracetam different from other antiepileptic drugs? Levetiracetam and its cellular mechanism of action in epilepsy revisited. Ther Adv Neurol Disord (2008) 1(1):13–24 10.1177/1756285608094212 21180561 PMC3002539

[2] Lynch BA Lambeng N Nocka K Kensel-Hammes P Bajjalieh SM Matagne A The synaptic vesicle protein SV2A is the binding site for the antiepileptic drug levetiracetam. Proc Natl Acad Sci U S A (2004) 101(26):9861–6 10.1073/pnas.0308208101 15210974 PMC470764

[3] Vogl C Mochida S Wolff C Whalley BJ Stephens GJ The synaptic vesicle glycoprotein 2A ligand levetiracetam inhibits presynaptic Ca^2+^ channels through an intracellular pathway. Mol Pharmacol (2012) 82(2):199–208 10.1124/mol.111.076687 22554805

[4] Yan HD Ishihara K Seki T Hanaya R Kurisu K Arita K Inhibitory effects of levetiracetam on the high-voltage-activated L-type Ca^2+^ channels in hippocampal CA3 neurons of spontaneously epileptic rat (SER). Brain Res Bull (2013) 90:142–8 10.1016/j.brainresbull.2012.10.006 23107646

[5] Lee DS Ryu HJ Kim JE Choi HC Kim YI Song HK The effect of levetiracetam on status epilepticus-induced neuronal death in the rat hippocampus. Seizure (2013) 22(5):368–77 10.1016/j.seizure.2013.02.005 23490457

[6] Gibbs JE Walker MC Cock HR Levetiracetam: antiepileptic properties and effects on mitochondrial dysfunction in experimental status epilepticus. Epilepsia (2006) 47(3):469–78 10.1111/j.1528-1167.2006.00454.x 16529608

[7] Gibbs JE Cock HR Administration of levetiracetam after prolonged status epilepticus does not protect from mitochondrial dysfunction in a rodent model. Epilepsy Res (2006) 73:208–12 10.1016/j.eplepsyres.2006.09.005 17085017

[8] Ueda Y Doi T Nagatomo K Tokumaru J Takaki M Willmore LJ Effect of levetiracetam on molecular regulation of hippocampal glutamate and GABA transporters in rats with chronic seizures induced by amygdalar FeCl_3_ injection. Brain Res (2007) 1151:55–61 10.1016/j.brainres.2007.03.021 17408599

[9] Haghikia A Ladage K Hinkerohe D Vollmar P Heupel K Dermietzel R Implications of antiinflammatory properties of the anticonvulsant drug levetiracetam in astrocytes. J Neurosci Res (2008) 86(8):1781–8 10.1002/jnr.21639 18335543

[10] Kim JE Choi HC Song HK Jo SM Kim DS Choi SY Levetiracetam inhibits interleukin-1 beta inflammatory responses in the hippocampus and piriform cortex of epileptic rats. Neurosci Lett (2010) 471(2):94–9 10.1016/j.neulet.2010.01.018 20080147

[11] Stienen MN Haghikia A Dambach H Thöne J Wiemann M Gold R Anti-inflammatory effects of the anticonvulsant drug levetiracetam on electrophysiological properties of astroglia are mediated via TGFβ1 regulation. Br J Pharmacol (2011) 162(2):491–507 10.1111/j.1476-5381.2010.01038.x 20955362 PMC3031068

[12] Li G Nowak M Bauer S Schlegel K Stei S Allenhöfer L Levetiracetam but not valproate inhibits function of CD8+ T lymphocytes. Seizure (2013) 22(6):462–6 10.1016/j.seizure.2013.03.006 23639870

[13] Ben-Menachem E Falter U Efficacy and tolerability of levetiracetam 3000 mg/d in patients with refractory partial seizures: a multicenter, double-blind, responder-selected study evaluating monotherapy. European levetiracetam Study Group. Epilepsia (2000) 41(10):1276–83 10.1111/j.1528-1157.2000.tb04605.x 11051122

[14] Cereghino JJ Biton V Abou-Khalil B Dreifuss F Gauer LJ Leppik I Levetiracetam for partial seizures: results of a double-blind, randomized clinical trial. Neurology (2000) 55(2):236–42 10.1212/WNL.55.2.236 10908898

[15] Betts T Waegemans T Crawford P A multicentre, double-blind, randomized, parallel group study to evaluate the tolerability and efficacy of two oral doses of levetiracetam, 2000 mg daily and 4000 mg daily, without titration in patients with refractory epilepsy. Seizure (2000) 9(2):80–7 10.1053/seiz.2000.0380 10845730

[16] Shorvon SD Löwenthal A Janz D Bielen E Loiseau P Multicenter double-blind, randomized, placebo-controlled trial of levetiracetam as add-on therapy in patients with refractory partial seizures. European levetiracetam Study Group. Epilepsia (2000) 41(9):1179–86 10.1111/j.1528-1157.2000.tb00323.x 10999557

[17] French J Edrich P Cramer JA A systematic review of the safety profile of levetiracetam: a new antiepileptic drug. Epilepsy Res (2001) 47(1-2):77–90 10.1016/S0920-1211(01)00296-0 11673023

[18] Harden C Safety profile of levetiracetam. Epilepsia (2001) 42(Suppl 4):36–9 10.1046/j.1528-1157.2001.00008.x 11564124

[19] Nguyen N Lee SB Lee YS Lee KH Ahn JY Neuroprotection by NGF and BDNF against neurotoxin-exerted apoptotic death in neural stem cells are mediated through Trk receptors, activating PI3-kinase and MAPK pathways. Neurochem Res (2009) 34(5):942–51 10.1007/s11064-008-9848-9 18846424

[20] Davidson WF Parish CR A procedure for removing red cells and dead cells from lymphoid cell suspensions. J Immunol Methods (1975) 7(2-3):291–300 10.1016/0022-1759(75)90026-5 167077

[21] Banki K Hutter E Gonchoroff NJ Perl A Elevation of mitochondrial transmembrane potential and reactive oxygen intermediate levels are early events and occur independently from activation of caspases in Fas signaling. J Immunol (1999) 162(3):1466–79 9973403 PMC4020419

[22] Waetzig V Loose K Haeusgen W Herdegen T Elevated Fas can be associated with neuronal differentiation (c-Jun N-terminal kinases mediate Fas-induced neurite regeneration in PC12 cells. Biochem Pharmacol (2008) 76(11):1476–84 10.1016/j.bcp.2008.07.014 18692025

[23] Kovács R Kardos J Heinemann U Kann O Mitochondrial calcium ion and membrane potential transients follow the pattern of epileptiform discharges in hippocampal slice cultures. J Neurosci (2005) 25(17):4260–9 10.1523/JNEUROSCI.4000-04.2005 15858052 PMC6725115

[24] Perry SW Norman JP Barbieri J Brown EB Gelbard HA Mitochondrial membrane potential probes and the proton gradient: a practical usage guide. Biotechniques (2011) 50(2):98–115 10.2144/000113610 21486251 PMC3115691

[25] Glancy B Balaban RS Role of mitochondrial Ca^2+^ in the regulation of cellular energetics. Biochemistry (2012) 51(14):2959–73 10.1021/bi2018909 22443365 PMC3332087

[26] Kwok KH Ho PW Chu AC Ho JW Liu HF Yiu DC Mitochondrial UCP5 is neuroprotective by preserving mitochondrial membrane potential, ATP levels, and reducing oxidative stress in MPP+ and dopamine toxicity. Free Radic Biol Med (2010) 49(6):1023–35 10.1016/j.freeradbiomed.2010.06.017 20600837

[27] Lang F Föller M Lang K Lang P Ritter M Vereninov A Cell volume regulatory ion channels in cell proliferation and cell death. Methods Enzymol (2007) 428:209–25 10.1016/S0076-6879(07)28011-5 17875419

[28] Desbarats J Birge RB Mimouni-Rongy M Weinstein DE Palerme JS Newell MK Fas engagement induces neurite growth through ERK activation and p35 upregulation. Nat Cell Biol (2003) 5(2):118–25 10.1038/ncb916 12545171

